# Distinguishing benign adult-onset mucosal pigmentation from syndromic lentigines: A diagnostic challenge

**DOI:** 10.1016/j.jdcr.2026.03.064

**Published:** 2026-04-13

**Authors:** Jane Hoban, Marion Leahy, Nicholas Stefanovic, Laoise Griffin

**Affiliations:** Department of Dermatology, University of Limerick Hospitals Group, University Hospital Limerick, Limerick, Ireland

**Keywords:** benign mucosal pigmentation, differential diagnosis, hamartomatous polyps, Laugier-Hunziker syndrome, longitudinal melanonychia, mucocutaneous pigmentation, Peutz-Jeghers syndrome, pigmentary disorders

## Case description

**Fig 1:**
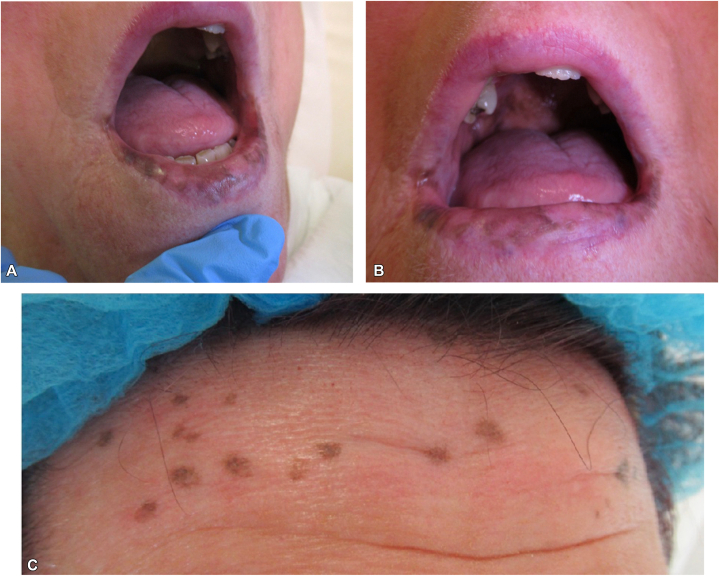
Laugier–Hunziker syndrome. **A** and **B,** Hyperpigmented macules involving the labial mucosa, gingiva, and hard palate. **C,** Atypical lentiginous macules on the forehead.

A 71-year-old Caucasian woman with no significant medical history presented with longstanding mucocutaneous hyperpigmented macules involving the labial mucosa, hard palate, and gingiva (Fig 1, *A* and *B*). The pigmentation was not present at birth or during childhood but developed gradually in adulthood and increased over time. There was no associated pain, bleeding, or ulceration. She reported longstanding mild alterations in bowel habit, including intermittent constipation and diarrhea. Her mother had exhibited similar mucosal pigmentation without gastrointestinal involvement. There was no personal or family history of gastrointestinal malignancy.

Examination revealed multiple well-demarcated brown to black macules affecting the oral mucosa. The nails demonstrated no longitudinal melanonychia or other pigmentation. Atypical lentiginous macules on the forehead (Fig 1, *C*) prompted biopsy. Histopathology showed increased basal layer pigmentation with focal pigment incontinence and no melanocytic atypia, excluding lentigo maligna.

Given the phenotypic overlap between benign and syndromic causes of mucocutaneous lentigines, and in the context of gastrointestinal symptoms, upper and lower endoscopy was performed. Three tubulovillous adenomas were identified in the caecum without hamartomatous polyps, dysplasia, or malignancy.

**Question: Which feature most strongly supports a diagnosis of Laugier–Hunziker syndrome over Peutz–Jeghers syndrome?**

A. Adult-onset progressive mucocutaneous pigmentation
B. Pathogenic mutation in *STK11*
C. Hamartomatous gastrointestinal polyps
D. Increased lifetime risk of pancreatic cancer
E. Autosomal dominant inheritance

Answer:

A. Adult-onset progressive mucocutaneous pigmentation.

## Discussion

The differential diagnosis of familial mucocutaneous lentigines is broad and includes Carney complex and LEOPARD syndrome; however, the absence of endocrine, cardiac, or other systemic abnormalities made these unlikely. In the absence of hamartomatous polyps or systemic involvement, and with adult-onset progressive pigmentation, Laugier–Hunziker syndrome (LHS) was favored.

Peutz–Jeghers syndrome (PJS) is a rare autosomal dominant disorder caused by pathogenic variants in the serine-threonine kinase gene (*STK11*), characterized by mucocutaneous lentigines and hamartomatous gastrointestinal polyps.^1^^,^^2^ It confers a substantially increased lifetime risk of malignancy involving the gastrointestinal tract, pancreas, breast, and reproductive organs.^3^ Identification of hamartomatous polyps or a pathogenic *STK11* mutation strongly supports PJS.

In contrast, LHS is a benign, idiopathic condition without known genetic association or systemic involvement. Although typically sporadic, rare familial cases have been reported.^4^ Both conditions share overlapping mucosal pigmentation, creating diagnostic uncertainty.

Longitudinal melanonychia is reported in approximately 40% to 60% of LHS cases^5^ and, when present, demonstrates homogeneous pigmentation without Hutchinson sign; however, its absence does not exclude the diagnosis, as nail involvement is not universal. Histopathologic findings in LHS characteristically demonstrate increased basal layer pigmentation without melanocytic atypia, as observed in this case.

The adult-onset progressive course further supports LHS, as lesions typically arise in early to mid-adulthood rather than being congenital. The absence of hamartomatous polyps and other syndromic features distinguishes LHS from PJS and other inherited lentiginosis syndromes.

Accurate distinction between mucocutaneous lentigines with familial or syndromic association and LHS is clinically imperative. PJS necessitates lifelong surveillance and genetic counselling due to malignancy risk, whereas LHS is entirely benign and does not require ongoing surveillance once syndromic causes have been excluded. When uncertainty exists, evaluation should be guided by clinical findings, histopathology, gastrointestinal symptoms, and genetic testing for pathogenic *STK11* variants where indicated.

This case underscores the importance of careful clinical correlation in distinguishing benign adult-onset mucocutaneous pigmentation from syndromic lentiginosis associated with systemic risk.

## Conflicts of interest

None disclosed.
